# Supplementary material for: PaECTER: Patent-level representation learning using citation-informed transformers
Source: PLoS One. 2026 Oct 27;21(10):e0358188. doi: 10.1371/journal.pone.0358188 (PMC13712717; doi:10.1371/journal.pone.0358188)
Supplement: Supplementary file 2 [file pone.0358188.s003.pdf]

Dear editor,
Dear Anonymous Referees,

On 13 July 2026, a minor revision was requested by the journal PLOS ONE. Unfortunately, no comments were passed on to the authors. Follow-on communication led to contradicting information. In any case, it is unclear what exactly the requested minor revision entails.

Hence we revised the manuscript, which had undergone 6 open peer-reviews for Computer Science conferences during 2024 and 205, in a minor way. The changes are detailed below.

For everything else we refer to our previous major revision from 1 July.

1. We have added few references. Notably, we include (1) and (2) to introduce the BM25 algorithm, and we quote (3) when we introduce the EPO's citation categories.

2. Furthermore, we have formatted several references (in-text vs. parenthesis).

References

[1] Robertson SE, Walker S. Some Simple Effective Approximations to the 2-Poisson Model for Probabilistic Weighted Retrieval. In: Sigir '94: Proceedings of the Seventeenth Annual International ACM-SIGIR Conference on Research and Development in Information Retrieval. Dublin City University: Springer London, Limited; 1994. .

[2] Robertson S, Zaragoza H. The Probabilistic Relevance Framework: BM25 and Beyond. Foundations and Trends in Information Retrieval. 2009 Sep;4(1-2):1-174. Available from: https://www.emerald.com/ftinr/article/4/1-2/1/1326508/The-Probabilistic-Relevance-Framework-BM25-and. http://dx.doi.org/10.1561/1500000019 doi:10.1561/1500000019.

[3] Wada T. When do the USPTO examiners cite as the EPO examiners? An analysis of examination spillovers through rejection citations at the international family-to-family level. Scientometrics. 2020 Nov;125(2):1591-615. Available from: https://link.springer.com/10.1007/s11192-020-03674-4. http://dx.doi.org/10.1007/s11192-020-03674-4 doi:10.1007/s11192-020-03674-4.
