# Supplementary material for: PaECTER: Patent-level representation learning using citation-informed transformers
Source: PLoS One. 2026 Oct 27;21(10):e0358188. doi: 10.1371/journal.pone.0358188 (PMC13712717; doi:10.1371/journal.pone.0358188)
Supplement: Peer review history

# Peer review: Decision Letter 0

**DOI:** 10.1371/journal.pone.0358188.r001

**Authors:** Federico Zilia

21 May 2026

Dear Dr. Rose,

Thank you for submitting your manuscript to PLOS ONE. After careful consideration, we feel that it has merit but does not fully meet PLOS ONE’s publication criteria as it currently stands. Therefore, we invite you to submit a revised version of the manuscript that addresses the points raised during the review process.

Please submit your revised manuscript by Jul 05 2026 11:59PM. If you will need more time than this to complete your revisions, please reply to this message or contact the journal office at plosone@plos.org. When you're ready to submit your revision, log on to https://www.editorialmanager.com/pone/ and select the 'Submissions Needing Revision' folder to locate your manuscript file.

- A letter that responds to each point raised by the academic editor and reviewer(s). You should upload this letter as a separate file labeled 'Response to Reviewers'.
- A marked-up copy of your manuscript that highlights changes made to the original version. You should upload this as a separate file labeled 'Revised Manuscript with Track Changes'.
- An unmarked version of your revised paper without tracked changes. You should upload this as a separate file labeled 'Manuscript'.

If you would like to make changes to your financial disclosure, please include your updated statement in your cover letter. Guidelines for resubmitting your figure files are available below the reviewer comments at the end of this letter.

If applicable, we recommend that you deposit your laboratory protocols in protocols.io to enhance the reproducibility of your results. Protocols.io assigns your protocol its own identifier (DOI) so that it can be cited independently in the future. For instructions see: https://journals.plos.org/plosone/s/submission-guidelines#loc-laboratory-protocols. Additionally, PLOS ONE offers an option for publishing peer-reviewed Lab Protocol articles, which describe protocols hosted on protocols.io. Read more information on sharing protocols at https://plos.org/protocols?utm_medium=editorial-email&utm_source=authorletters&utm_campaign=protocols.

As the corresponding author, your ORCID iD is verified in the submission system and will appear in the published article. PLOS supports the use of ORCID, and we encourage all coauthors to register for an ORCID iD and use it as well. Please encourage your coauthors to verify their ORCID iD within the submission system before final acceptance, as unverified ORCID iDs will not appear in the published article. *Only*  the individual author can complete the verification step; PLOS staff *cannot*  verify ORCID iDs on behalf of authors.

We look forward to receiving your revised manuscript.

Kind regards,

Federico Zilia

Academic Editor

PLOS One

Journal Requirements:

When submitting your revision, we need you to address these additional requirements.

1. Please ensure that your manuscript meets PLOS ONE's style requirements, including those for file naming. The PLOS ONE style templates can be found at

https://journals.plos.org/plosone/s/file?id=wjVg/PLOSOne_formatting_sample_main_body.pdf and

https://journals.plos.org/plosone/s/file?id=ba62/PLOSOne_formatting_sample_title_authors_affiliations.pdf

2. Please note that PLOS One has specific guidelines on code sharing for submissions in which author-generated code underpins the findings in the manuscript. In these cases, we expect all author-generated code to be made available without restrictions upon publication of the work. Please review our guidelines at https://journals.plos.org/plosone/s/materials-and-software-sharing#loc-sharing-code and ensure that your code is shared in a way that follows best practice and facilitates reproducibility and reuse.

3. Thank you for stating the following financial disclosure: [Our work has been enabled by grant 2023/8401 from the European Patent Office under their Academic Research Programme (EPO-ARP) 2021.].

Please state what role the funders took in the study.  If the funders had no role, please state: ""The funders had no role in study design, data collection and analysis, decision to publish, or preparation of the manuscript.""

If this statement is not correct you must amend it as needed.

Please include this amended Role of Funder statement in your cover letter; we will change the online submission form on your behalf.

4. Please note that your Data Availability Statement is currently missing the DOI/accession number of each dataset OR a direct link to access each database]. If your manuscript is accepted for publication, you will be asked to provide these details on a very short timeline. We therefore suggest that you provide this information now, though we will not hold up the peer review process if you are unable.

5. Please amend your authorship list in your manuscript file to include author Dr. Erik Buunk.

6. If the reviewer comments include a recommendation to cite specific previously published works, please review and evaluate these publications to determine whether they are relevant and should be cited. There is no requirement to cite these works unless the editor has indicated otherwise.

7. Please review your reference list to ensure that it is complete and correct. If you have cited papers that have been retracted, please include the rationale for doing so in the manuscript text, or remove these references and replace them with relevant current references. Any changes to the reference list should be mentioned in the rebuttal letter that accompanies your revised manuscript. If you need to cite a retracted article, indicate the article’s retracted status in the References list and also include a citation and full reference for the retraction notice.

[Note: HTML markup is below. Please do not edit.]

Reviewers' comments:

Reviewer's Responses to Questions

**Comments to the Author**

1. Is the manuscript technically sound, and do the data support the conclusions?

Reviewer #1: Partly

Reviewer #2: Yes

**********

2. Has the statistical analysis been performed appropriately and rigorously?

Reviewer #1: Yes

Reviewer #2: Yes

**********

3. Have the authors made all data underlying the findings in their manuscript fully available?

The PLOS Data policy

Reviewer #1: Yes

Reviewer #2: Yes

**********

4. Is the manuscript presented in an intelligible fashion and written in standard English?

Reviewer #1: Yes

Reviewer #2: Yes

**********

Reviewer #1: The manuscript presents PaECTER, a BERT-based model for patent representation learning trained via contrastive learning using citation data. The authors release the model and the training dataset open-source, which is a valuable contribution to the field. While the results on citation prediction tasks appear promising, I have identified specific concerns regarding the data splitting methodology and the conceptual validity of using abstracts for prior art search tasks that need to be addressed to ensure the technical soundness of the research.

++ Major Comments ++

- Risk of data leakage via patent families

The authors note that "none of the focal, negative, or positive patent selections are present in the training dataset". In the patent domain, however, excluding individual document IDs is not sufficient because patents are organised in families (eg DOCDB / INPADOC). If one family member (for instance, a US application) appears in the training set while another family member with the same priority, title, and near-identical abstract (eg an EP equivalent) appears in the test set, the evaluation does not reflect true generalization. Rather, the model may simply be recalling previously seen content. For this reason, it is important to clarify explicitly in Section 2.5 whether the Train/Validation/Test split was performed at the patent family level. In other words, if a given family is included in the training set, no member of that family should appear in the validation or test sets. If this was not the case, the reported performance metrics are likely to be inflated, and the data split should be reconsidered accordingly.

- Input limitation (abstract vs claims) and task validity

The model is trained on Title + Abstract, truncated to 512 tokens. While this choice is understandable from a computational perspective, it represents a strong limitation given the stated application of prior art search for patent examiners. In practice, a patent’s legal scope and technical novelty are defined by the claims, whereas abstracts are often deliberately broad and sometimes strategically uninformative. In this light, it is not immediately intuitive that PaECTER (abstract-based) outperforms SEARCHFORMER (claim-based), even on SEARCHFORMER's own benchmark. The manuscript would benefit from a clearer discussion of why abstracts are sufficient for the specific prediction task addressed here. For instance, does the evaluation setup favour broad semantic similarity rather than precise matching of technical features? More generally, the limitations of abstract-based representations for applications such as novelty assessment or Freedom-to-Operate analysis should be acknowledged and discussed more transparently in the Discussion section.

- Comparison with general-purpose LLM embeddings

Table 2 shows that general-purpose embedding models such as BGE-large-en-v1.5 achieve performance levels close to PaECTER (e.g. MRR@10 of 81.18 versus 88.25). While PaECTER remains superior, the margin is not large, and in the external evaluation (Section 4.3) general models such as GTE or S-BERT occasionally outperform PaECTER in classification tasks. This raises the question of whether PaECTER may be somewhat specialised, or even overfittedd, to the citation prediction task itself (i.e. learning "citability" patterns), potentially at the expense of broader semantic understanding that general models capture more effectively. A short discussion addressing this trade-off would strengthen the interpretation of the results and better position the contribution of the model.

++ Minor Comments ++

- Definition of hard negatives

Hard negatives are defined as patents cited by the focal patent's backward citations but not cited by the focal patent itself. Did the authors verify that these documents are not cited as category "A" (technological background) in parallel proceedings or in other members of the same patent family? In some cases, so-called hard negatives may actually represent valid semantic matches, effectively becoming false negatives in a contrastive learning setup. Clarifying how this risk was handled would help interpret the training strategy more accurately.

- Evaluation metrics

The evaluation relies on MRR, MAP, and RFR. Could the authors explain why nDCG (Normalized Discounted Cumulative Gain) was not included? nDCG is a standard metric in Information Retrieval and is particularly well suited to ranking tasks where the position of relevant documents matters.

- Tokenisation

Since the model builds on "BERT for Patents", it would be informative to report the average number of [UNK] tokens compared to a standard BERT tokenizer. This would provide a quantitative justification for the use of a patent-specific vocabulary and strengthen the methodological motivation.

Reviewer #2: The paper presents PaECTER, a citation-informed patent representation model with strong performance in similarity and citation prediction tasks. The methodology is relevant and experimentally validated against existing models. The work is suitable for publication after minor revision. Authors should include a more updated literature review covering recent transformer-based patent retrieval and semantic search approaches.

**********

what does this mean?). If published, this will include your full peer review and any attached files.

If you choose “no”, your identity will remain anonymous but your review may still be made public.

**Do you want your identity to be public for this peer review?**  For information about this choice, including consent withdrawal, please see our Privacy Policy

Reviewer #1: No

Reviewer #2: No

**********

[NOTE: If reviewer comments were submitted as an attachment file, they will be attached to this email and accessible via the submission site. Please log into your account, locate the manuscript record, and check for the action link "View Attachments". If this link does not appear, there are no attachment files.]

To ensure your figures meet our technical requirements, please review our figure guidelines: https://journals.plos.org/plosone/s/figures

You may also use PLOS’s free figure tool, NAAS, to help you prepare publication quality figures: https://journals.plos.org/plosone/s/figures#loc-tools-for-figure-preparation.

NAAS will assess whether your figures meet our technical requirements by comparing each figure against our figure specifications.

# Author comment: Author response to Decision Letter 1

**DOI:** 10.1371/journal.pone.0358188.r002

2 Jul 2026

Dear editor,

Dear Anonymous Referees,

We would like to thank you for your helpful comments and suggestions in this revision process. We respond to the concerns and suggestions one-by-one below.

Reviewer #1:

1. The manuscript presents PaECTER, a BERT-based model for patent representation learning trained via contrastive learning using citation data. The authors release the model and the training dataset open-source, which is a valuable contribution to the field. While the results on citation prediction tasks appear promising, I have identified specific concerns regarding the data splitting methodology and the conceptual validity of using abstracts for prior art search tasks that need to be addressed to ensure the technical soundness of the research.

Thank you very much for taking the time to read our manuscript and provide extensive commentary!

2. - Risk of data leakage via patent families

The authors note that "none of the focal, negative, or positive patent selections are present in the training dataset". In the patent domain, however, excluding individual document IDs is not sufficient because patents are organised in families (eg DOCDB / INPADOC). If one family member (for instance, a US application) appears in the training set while another family member with the same priority, title, and near-identical abstract (eg an EP equivalent) appears in the test set, the evaluation does not reflect true generalization. Rather, the model may simply be recalling previously seen content. For this reason, it is important to clarify explicitly in Section 2.5 whether the Train/Validation/Test split was performed at the patent family level. In other words, if a given family is included in the training set, no member of that family should appear in the validation or test sets. If this was not the case, the reported performance metrics are likely to be inflated, and the data split should be reconsidered accordingly.

You raise an important concern here. We agree that occasionally scholars ignore patent families when counting patents or when measuring semantic similarity. However, our entire training set consists exclusively of granted EP patents. (Notably, there are no USPTO-issued documents in our datasets.) Within the EPO, DocDB families might still arise due to divisionals, though these are rare (Harhoff (2016). In our dataset, we have deduplicated every document at the DocDB level. Additionally, we have deduplicated the training and the validation sets based on text (title concatenated with abstract) which we didn't report earlier. To address this concern, we have amended a paragraph in section 2.2:

"In total, 1,358,264 patent documents satisfy these criteria. From those, we randomly sample 300,000 documents after deduplicating based on text (title concatenated with abstract)." This is followed by the new footnote 4: "Since all our focal patents are granted EP patents, multiple patents belonging to the same DocDB family may arise especially due to divisional patent filings (Harhoff, 2016). No DocDB family exists more than once in our dataset though."

3. - Input limitation (abstract vs claims) and task validity

The model is trained on Title + Abstract, truncated to 512 tokens. While this choice is understandable from a computational perspective, it represents a strong limitation given the stated application of prior art search for patent examiners. In practice, a patent’s legal scope and technical novelty are defined by the claims, whereas abstracts are often deliberately broad and sometimes strategically uninformative. In this light, it is not immediately intuitive that PaECTER (abstract-based) outperforms SEARCHFORMER (claim-based), even on SEARCHFORMER's own benchmark. The manuscript would benefit from a clearer discussion of why abstracts are sufficient for the specific prediction task addressed here. For instance, does the evaluation setup favour broad semantic similarity rather than precise matching of technical features? More generally, the limitations of abstract-based representations for applications such as novelty assessment or Freedom-to-Operate analysis should be acknowledged and discussed more transparently in the Discussion section.

Thank you for sharing these thoughts! Indeed, the 512-token limitation is problematic. However, it reflects a pragmatic tradeoff made by the BERT developers. We also agree that a patent's legal scope is defined by its claims. However, patent abstracts are widely used in the literature to summarise inventions. Examples include Kelly et al. (2021), Balsmeier et al. (2018) or Arts et al. (2021). Recently, Chikkamath et al. (2026) show that embeddings from patent abstracts often (not always) perform better than embeddings from claims. On the other hand, PaECTER is agnostic to the input data; therefore nothing stops users from applying PaECTER on claims or patent full-texts.

To put the relative performance differentials into perspective, we have amended section 4.2 as follows:

"That PaECTER outperforms SEARCHFORMER may reflect different training strategies. SEARCHFORMER uses claims as a proxy for citation prediction, whereas PaECTER uses titles and abstracts but for the same purpose. While patent claims define the legal scope of protection and are central to novelty assessments, our evaluation task differs from a full patentability assessment. Both our benchmark and the SEARCHFORMER benchmark evaluate citation prediction through textual similarity, rather than detailed claim interpretation. PaECTER’s stronger performance relative to SEARCHFORMER suggests that titles and abstracts contain sufficient information to capture many of the semantic signals underlying examiner citation decisions, making them effective for identifying technologically related prior art. More specifically, our evaluation setup rewards the retrieval of relevant and technologically related patents rather than the precise matching of technical features embodied in patent claims. PaECTER appears particularly well suited for the former, where technological relevance is the primary objective.

At the same time, this finding should not be interpreted as evidence that claims are unnecessary for patent examination or prior-art analysis. Applications requiring legal scrutiny, novelty assessments, inventive-step evaluations, or Freedom-to-Operate analyses are likely to benefit from incorporating claims and potentially the full patent specification in a more extensive exercise."

4. - Comparison with general-purpose LLM embeddings

Table 2 shows that general-purpose embedding models such as BGE-large-en-v1.5 achieve performance levels close to PaECTER (e.g. MRR@10 of 81.18 versus 88.25). While PaECTER remains superior, the margin is not large, and in the external evaluation (Section 4.3) general models such as GTE or S-BERT occasionally outperform PaECTER in classification tasks. This raises the question of whether PaECTER may be somewhat specialised, or even overfittedd, to the citation prediction task itself (i.e. learning "citability" patterns), potentially at the expense of broader semantic understanding that general models capture more effectively. A short discussion addressing this trade-off would strengthen the interpretation of the results and better position the contribution of the model.

Thank you for bringing this up. However, we disagree that the margin is not large: The second-best performing model (BGE-large) has a MAP of 60.17 and an MRR@10 of 81.19, while PaECTER achieves 68.17 and 88.25 in these metrics. That is a substantial improvement.

When it comes to the external evaluation you are correct. Ganguli et al. (2024) have benchmarked PaECTER against other models on tasks that PaECTER wasn't trained for, such as classification or historic data. Yet it is clear that PaECTER is great at inference tasks.

To clarify the limitations and PaECTER's purpose, we have amended the conclusion (because this trade-off is not only informed by the external evaluation): "PaECTER's main task is that of a citation recommender, i.e., it excels at retrieving related patent literature for instance during the examination process. Though PaECTER can in principle be used for a variety of tasks (semantic distance, classification/clustering, etc.), we recommend users to benchmark several models on these tasks."

5. - Definition of hard negatives

Hard negatives are defined as patents cited by the focal patent's backward citations but not cited by the focal patent itself. Did the authors verify that these documents are not cited as category "A" (technological background) in parallel proceedings or in other members of the same patent family? In some cases, so-called hard negatives may actually represent valid semantic matches, effectively becoming false negatives in a contrastive learning setup. Clarifying how this risk was handled would help interpret the training strategy more accurately.

Thank you for raising this concern. In our set-up we have relied exclusively on patent grants by the EPO for two reasons: Citations are added only by examiners and citations fall into categories. We know of no other patent authority where this applies. Thus, other members of the same DocDB family from another patent authority will not provide this citation classification system. The question of whether a patent is cited as "A" by one member of a DocDB family but not by its sibling can therefore only apply to two EPO patents. The only way a DocDB family enters our dataset twice is through divisional patent applications, which are rare (see Harhoff (2016)). In fact, in our dataset used for training and validation, this happens 7,671 times (out of 300k (2.5%). In several cases, however, the sibling patent does not cite anything, reducing this set to 6,250 families.

To introduce the case that an EPO divisional of a focal patent (also EPO) cites a document that the focal patent doesn't cite, these two documents must have different search reports. That happens 4,720 (1.5%) times. Based on a direct comparison between the sibling citations (A/X/Y) and the citations in our training/validation data set, we found that in 30 cases a sibling citation was used as a hard negative (out of 3 million negative pairs). We have thus added this in a footnote 5: "In retrospect, we discovered that 30 hard negatives were positive citations of patent siblings. These patent siblings were divisional filings, which are rare at the EPO (Harhoff, 2016)."

Thank you again for raising this interesting concern that we were not aware of. A future version of PaECTER will take this into account.

5. - Evaluation metrics

The evaluation relies on MRR, MAP, and RFR. Could the authors explain why nDCG (Normalized Discounted Cumulative Gain) was not included? nDCG is a standard metric in Information Retrieval and is particularly well suited to ranking tasks where the position of relevant documents matters.

Thank you for this question and suggestion. We had initially omitted nDCG because our (positive or negative) samples are not ranked within groups. However, we have come to know that nDCG can also be computed for binary outcomes, thus we have added results for this measure to Table 2 along with the following sentence (in section 4.1):

"Thirdly, we use Normalized Discounted Cumulative Gain @ 10 (nDCG@10), which, treating each candidate as simply relevant or non-relevant, measures the discounted cumulative gain of the top 10 ranked patents normalized against that of an ideal ranking, averaged across all test samples."

We find that also according to this metric, PaECTER outperforms the other models.

6. - Tokenisation

Since the model builds on "BERT for Patents", it would be informative to report the average number of [UNK] tokens compared to a standard BERT tokenizer. This would provide a quantitative justification for the use of a patent-specific vocabulary and strengthen the methodological motivation.

Thank you for this suggestion! We have computed the number of abstracts that contain [UNK] tokens in our dataset both for BERT (plain vanilla) and PaECTER. BERT finds 1,743 abstracts with [UNK] tokens, and PaECTER exactly 10 more. This amounts to less than 0.01% of all samples used. We report on this in the new footnote 6: "Within our text corpus, 1,753 abstracts contain [UNK] tokens using BERT for Patents. For comparison, the number of abstracts with [UNK] tokens using BERT is 1,743."

We also report on the number of truncated abstracts in the following footnote 7, which is 4,895 (0.25%): "In our corpus, the mean number of tokens per document is 159 with 4,895 documents (0.25%) being above the 512 token limit."

Reviewer #2

1. The paper presents PaECTER, a citation-informed patent representation model with strong performance in similarity and citation prediction tasks. The methodology is relevant and experimentally validated against existing models. The work is suitable for publication after minor revision. Authors should include a more updated literature review covering recent transformer-based patent retrieval and semantic search approaches.

Thank you for your assessment! First, we have updated the references to studies that we already cite (preprint to published version). Second, we now also include subsequent developments in the area of patent retrieval. In the introduction, we write:

"Since PaECTER's release, several studies have benchmarked against PaECTER on a variety of tasks. We report on the benchmarking exercises of Ganguli et al. (2024) and Ayaou and Cavallucci (2025) in section 4.3.

There is also a growing body of work that provides alternative models for patent ranking and retrieval. We summarize these developments to situate our contribution within the current landscape rather than to claim priority over later efforts. Recent model developments include the patembed model family by Ayaou and Cavallucci (2025), the unbranded model by Daniell et al. (2025) as well as the PAI-NET by Lee and Bai (2025). PAI-NET (Lee and Bai, 2025) also uses a contrastive learning approach, but trains on examiner-added patent citations in patents granted by the USPTO or the KIPO. The patembed family (Ayaou and Cavallucci, 2025) is also built on BERT for Patents but is trained jointly across multiple retrieval and classification tasks and extends the input context window. The model of Daniell et al. (2025) departs from text encoders altogether, as they represent each patent document as a graph of features and their relationships that a Graph Transformer embeds for dense retrieval."

We also include patembed-large in our test suite; the other aforementioned models (PAI-NET, the Daniell et al. model) were not available. Table 2 has been updated accordingly. We find that patembed-large performs second when prompted for same document retrieval. The updated Table 2 is shown in the attached PDF.

Finally, in section 4.3, we discuss the performance in the PatenTEB suit specifically:

"Ayaou and Cavallucci (2025) have also tested PaECTER, among other models, as a baseline in their PatenTEB benchmark, a suite of 15 patent-specific tasks constructed from roughly 2.06 million examples drawn from multi-jurisdictional patent families. The benchmark groups the tasks into four families: retrieval, classification, paraphrase detection, and clustering. Other models include the authors' own patembed family, general-purpose encoders such as GTE-ModernBERT and Qwen3-Embedding, and patent-specialized baselines such as PatentSBERTa and BERT for Patents.

The retrieval family comprises eight tasks: three symmetric document-to-document tasks (same-domain, mixed-domain, and cross-domain) in which relevance is defined by citation links, and five asymmetric fragment-to-document tasks (title-to-full, problem-to-full, problem-to-solution, effect-to-full, and effect-to-substance) that query full documents using only a fragment. Performance is measured by NDCG@10, a rank-aware metric. The three symmetric tasks are the ones closest to PaECTER's own training objective of citation-supervised prior-art retrieval, and on these PaECTER is the strongest model after the purpose-built patembed family, ahead of both general-purpose encoders (e.g., 0.421 vs. 0.395 for Qwen3 and 0.363 for GTE-ModernBERT on the same-domain task). On the asymmetric tasks, which lie outside its design, PaECTER trails the general encoders, most clearly on title-to-full (0.454 against 0.689--0.738).

The classification family covers three tasks: assigning patents to one of 109 IPC3 technology classes, predicting a patent's citation-timing trajectory, and predicting the direction of a citation between two patents. Performance is measured by the macro-F1. Here PaECTER averages 0.536, resulting in a third place. This reflects that its document-level embeddings capture technological domain information well even though the model was not trained for classification.

The paraphrase family consists of two tasks that test whether the model recognizes problem or solution segments as semantically equivalent, with positive pairs sampled from same-domain citation pairs. Performance is measured by the Pearson correlation coefficient. PaECTER reaches 0.753 on these tasks, below the patembed family and somewhat above the general encoders.

The clustering family comprises two evaluation-only tasks that group patent families by their full set of IPC codes and by inventor identity. Performance is measured by V-measure. PaECTER scores 0.621, among the best of all evaluated models and slightly above patembed-large. This result is somewhat surprising given that clustering is a task it was never trained for."

Thank you again for the opportunity to improve our manuscript, and we look forward to hearing back from you.

Sincerely,

The authors

[Table and References in the attached PDF.]

Attachment Submitted filename: response.pdf

# Editor report: Decision Letter 1

**DOI:** 10.1371/journal.pone.0358188.r003

**Authors:** Federico Zilia

12 Jul 2026

PONE-D-25-60240R1

PaECTER: Patent-level Representation Learning using Citation-informed Transformers

PLOS One

Dear Dr. Rose,

Thank you for submitting your manuscript to PLOS ONE. After careful consideration, we feel that it has merit but does not fully meet PLOS ONE’s publication criteria as it currently stands. Therefore, we invite you to submit a revised version of the manuscript that addresses the points raised during the review process.

================================

Both reviewers highlight some minor concerns that must be addressed before the manuscript can be considered for publication.

================================

Please submit your revised manuscript by Aug 26 2026 11:59PM. If you will need more time than this to complete your revisions, please reply to this message or contact the journal office at plosone@plos.org. When you're ready to submit your revision, log on to https://www.editorialmanager.com/pone/ and select the 'Submissions Needing Revision' folder to locate your manuscript file.

Please include the following items when submitting your revised manuscript:

- A letter that responds to each point raised by the academic editor and reviewer(s). You should upload this letter as a separate file labeled 'Response to Reviewers'.
- A marked-up copy of your manuscript that highlights changes made to the original version. You should upload this as a separate file labeled 'Revised Manuscript with Track Changes'.
- An unmarked version of your revised paper without tracked changes. You should upload this as a separate file labeled 'Manuscript'.

If you would like to make changes to your financial disclosure, please include your updated statement in your cover letter. Guidelines for resubmitting your figure files are available below the reviewer comments at the end of this letter.

If applicable, we recommend that you deposit your laboratory protocols in protocols.io to enhance the reproducibility of your results. Protocols.io assigns your protocol its own identifier (DOI) so that it can be cited independently in the future. For instructions see: https://journals.plos.org/plosone/s/submission-guidelines#loc-laboratory-protocols. Additionally, PLOS ONE offers an option for publishing peer-reviewed Lab Protocol articles, which describe protocols hosted on protocols.io. Read more information on sharing protocols at https://plos.org/protocols?utm_medium=editorial-email&utm_source=authorletters&utm_campaign=protocols.

As the corresponding author, your ORCID iD is verified in the submission system and will appear in the published article. PLOS supports the use of ORCID, and we encourage all coauthors to register for an ORCID iD and use it as well. Please encourage your coauthors to verify their ORCID iD within the submission system before final acceptance, as unverified ORCID iDs will not appear in the published article. *Only*  the individual author can complete the verification step; PLOS staff *cannot*  verify ORCID iDs on behalf of authors.

We look forward to receiving your revised manuscript.

Kind regards,

Federico Zilia

Academic Editor

PLOS One

Journal Requirements:

If the reviewer comments include a recommendation to cite specific previously published works, please review and evaluate these publications to determine whether they are relevant and should be cited. There is no requirement to cite these works unless the editor has indicated otherwise.

Please review your reference list to ensure that it is complete and correct. If you have cited papers that have been retracted, please include the rationale for doing so in the manuscript text, or remove these references and replace them with relevant current references. Any changes to the reference list should be mentioned in the rebuttal letter that accompanies your revised manuscript. If you need to cite a retracted article, indicate the article’s retracted status in the References list and also include a citation and full reference for the retraction notice.

[Note: HTML markup is below. Please do not edit.]

[NOTE: If reviewer comments were submitted as an attachment file, they will be attached to this email and accessible via the submission site. Please log into your account, locate the manuscript record, and check for the action link "View Attachments". If this link does not appear, there are no attachment files.]

To ensure your figures meet our technical requirements, please review our figure guidelines: https://journals.plos.org/plosone/s/figures

You may also use PLOS’s free figure tool, NAAS, to help you prepare publication quality figures: https://journals.plos.org/plosone/s/figures#loc-tools-for-figure-preparation.

NAAS will assess whether your figures meet our technical requirements by comparing each figure against our figure specifications.

# Author comment: Author response to Decision Letter 2

**DOI:** 10.1371/journal.pone.0358188.r004

25 Aug 2026

In this minor revision, there were none passed to us. Please refer to the document response.pdf or to the response from the previous major revision dating 1 July.

Attachment Submitted filename: response_auresp_2.pdf

# Editor report: Decision Letter 2

**DOI:** 10.1371/journal.pone.0358188.r005

**Authors:** Federico Zilia

27 Aug 2026

PaECTER: Patent-level Representation Learning using Citation-informed Transformers

PONE-D-25-60240R2

Dear Dr. Michael E. Rose,

We’re pleased to inform you that your manuscript has been judged scientifically suitable for publication and will be formally accepted for publication once it meets all outstanding technical requirements.

Within one week, you’ll receive an e-mail detailing the required amendments. When these have been addressed, you’ll receive a formal acceptance letter and your manuscript will be scheduled for publication.

An invoice will be generated when your article is formally accepted. Please note, if your institution has a publishing partnership with PLOS and your article meets the relevant criteria, all or part of your publication costs will be covered. Please make sure your user information is up-to-date by logging into Editorial Manager at Editorial Manager® and clicking the ‘Update My Information' link at the top of the page. For questions related to billing, please contact billing support.

If your institution or institutions have a press office, please notify them about your upcoming paper to help maximize its impact. If they’ll be preparing press materials, please inform our press team as soon as possible -- no later than 48 hours after receiving the formal acceptance. Your manuscript will remain under strict press embargo until 2 pm Eastern Time on the date of publication. For more information, please contact onepress@plos.org.

Kind regards,

Federico Zilia

Academic Editor

PLOS One

Additional Editor Comments (optional):

Reviewers' comments:

# Editor report: Acceptance letter

**DOI:** 10.1371/journal.pone.0358188.r006

**Authors:** Federico Zilia

PONE-D-25-60240R2

PLOS One

Dear Dr. Rose,

I'm pleased to inform you that your manuscript has been deemed suitable for publication in PLOS One. Congratulations! Your manuscript is now being handed over to our production team.

At this stage, our production department will prepare your paper for publication. This includes ensuring the following:

* All references, tables, and figures are properly cited

* All relevant supporting information is included in the manuscript submission,

* There are no issues that prevent the paper from being properly typeset

You will receive further instructions from the production team, including instructions on how to review your proof when it is ready. Please keep in mind that we are working through a large volume of accepted articles, so please give us a few days to review your paper and let you know the next and final steps.

Lastly, if your institution or institutions have a press office, please let them know about your upcoming paper now to help maximize its impact. If they'll be preparing press materials, please inform our press team within the next 48 hours. Your manuscript will remain under strict press embargo until 2 pm Eastern Time on the date of publication. For more information, please contact onepress@plos.org.

You will receive an invoice from PLOS for your publication fee after your manuscript has reached the completed accept phase. If you receive an email requesting payment before acceptance or for any other service, this may be a phishing scheme. Learn how to identify phishing emails and protect your accounts at https://explore.plos.org/phishing.

If we can help with anything else, please email us at customercare@plos.org.

Thank you for submitting your work to PLOS One and supporting open access.

Kind regards,

PLOS One Editorial Office Staff

on behalf of

Dr. Federico Zilia

Academic Editor

PLOS One
